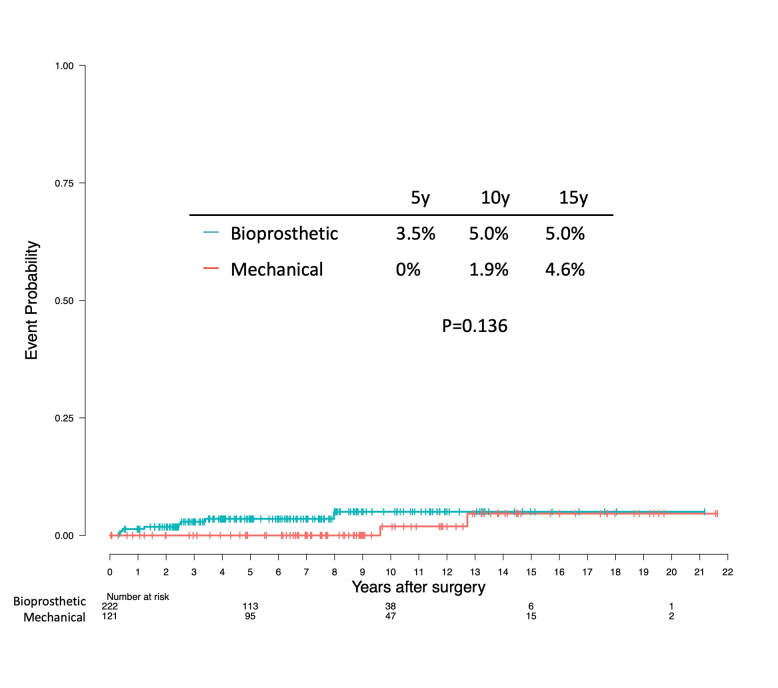# Long-term Outcomes After Surgical Aortic Valve Replacement in Patients ≤65 Years: Comparison of Age- and Sex-Matched Japanese General Population

**DOI:** 10.1016/j.atssr.2024.12.008

**Published:** 2025-01-03

**Authors:** Masaya Hirayama, Satoshi Kainuma, Katsuhiro Omae, Naonori Kawamoto, Kota Suzuki, Takashi Kakuta, Takeshi Kitai, Chisato Izumi, Satsuki Fukushima

**Affiliations:** 1 Department of Cardiovascular Surgery, National Cerebral and Cardiovascular Center, Osaka, Japan; 2 Department of Data Science, National Cerebral and Cardiovascular Center, Osaka, Japan; 3 Cardiovascular Medicine, National Cerebral and Cardiovascular Center, Osaka, Japan

## Abstract

**Background:**

With the widespread use of transcatheter aortic valve implantation, accurate assessment of the long-term outcomes of surgical aortic valve replacement has become more pertinent, particularly in young adults. The aim of this study was to clarify the long-term survival after surgical aortic valve replacement for patients aged ≤65 years compared with the age- and sex-matched Japanese general population.

**Methods:**

This study analyzed 343 patients, aged ≤65 years (mean age, 53.2 ± 10.5 years), who underwent primary surgical aortic valve replacement with bioprosthetic or mechanical prosthetic valves from 2001 to 2021. The overall survival rate after surgical aortic valve replacement was compared with an age- and sex-matched Japanese general population. The median follow-up period was 80 months (interquartile range, 43-125 months).

**Results:**

The overall 15-year survival rate was neither different from that of the Japanese general population (87.4% vs 90.0%, *P* = .899) nor significantly different when patients were stratified by age ≤60 years (88.8% vs 93.0%, log-rank *P* = .563) and ≤50 years (95.3% vs 97.0%, log-rank *P* = .657). The standardized mortality ratios in the entire cohort (aged ≤65 years) and in patients aged ≤60 and ≤50 years were 1.033 (95% CI, 0.584-1.829), 1.213 (95% CI, 0.584-2.518), and 1.367 (95% CI, 0.320-5.849), respectively.

**Conclusions:**

The survival rate after surgical aortic valve replacement in young adults aged ≤65 years was equivalent to that of the age- and sex-matched Japanese general population. Our data may help guide future therapeutic comparisons for patients aged ≤65 years requiring aortic valve procedures.

## Visual Abstract

**Figure:**
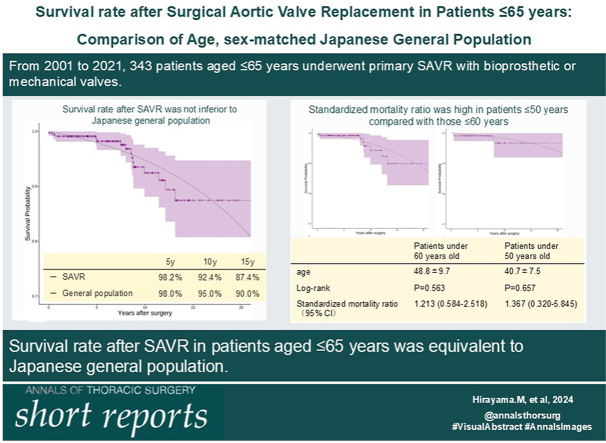

**In Short**

▪ The survival rate of young adults aged ≤65 years who underwent surgical aortic valve replacement was equivalent to that of the age- and sex-matched general Japanese population.
▪ The standardized mortality ratio tended to be high in younger patients at the time of the operation.
▪ Our data may help guide future therapeutic comparisons for patients aged ≤65 years requiring aortic valve procedures.

Surgical aortic valve replacement (SAVR) is the standard treatment for most young adults with severe aortic stenosis and/or regurgitation. However, treatment options have become diverse because of the improved durability of bioprosthetic valves, widespread use of transcatheter aortic valve implantation (TAVI),^1^ and revival of the Ross procedure in the United States.^2^, ^3^, ^4^, ^5^ Therefore, the optimal strategy for aortic valve surgery in young adults remains controversial. A trend toward the use of bioprosthetic valves has been recognized in younger patients in association with the availability of TAVI as a subsequent intervention for failed biological aortic valve replacement (AVR; valve-in-valve).^6^ In contrast, mechanical valves are more durable but require a lifetime of anticoagulation and lifestyle modifications.

In the United States, some studies recently suggested that the survival rate of the Ross procedure is better than that of SAVR with the use of either biological or mechanical valves and is comparable to that of the age-, sex-, and race-matched United States general population.^4^^,^^5^ This finding has attracted a lot of attention worldwide, and the aim of the current study was to clarify and compare the long-term survival after SAVR of patients aged ≤65 years against that of an age- and sex-matched Japanese general population.

## Patients and Methods

This nonrandomized retrospective study encompassed patients aged ≤65 years who underwent primary SAVR, with or without concomitant surgery, at our center between 2001 and 2021. The study complied with the principles outlined in the Declaration of Helsinki and was approved by the National Cerebral and Cardiovascular Center Institutional Review Board (reference number: M30-026). All patients provided informed consent before undergoing the procedures.

Patients who had a history of aortic valve surgery and required a second AVR or those with active infectious endocarditis were excluded. SAVR was performed with a bioprosthetic or mechanical prosthetic valve after careful discussion with the patients based on the Japanese guidelines on the management of valvular heart diseases. A detailed description of all methods involved in this study is provided in the Supplemental Material.

## Results

The baseline demographics of the 343 patients enrolled in the study are summarized in Table 1. In the entire cohort, the mean age was 53.2 ± 10.5 years, 224 patients (65%) were men, 56% presented with severe aortic stenosis, and the median preoperative left ventricular ejection fraction was 0.56. Clinical follow-up examinations were completed in 301 patients (87.8%), with a median follow-up period of 80.4 months (interquartile range, 43.2-124.5 months). The cumulative follow-up period was 2502 patient-years.

**Table 1:** Patient Characteristics

Variables	Total	Bioprosthetic Valve	Mechanical Valve	*P* Value
	(N = 343)	(n = 222)	(n = 121)	
Age, y	53.2 ± 10.5	55.5 ± 9.6	49.0 ± 11.0	<.001
Male sex	224 (65)	142 (64)	82 (68)	.553
Body surface area, m^2^	1.70 (1.53-1.84)	1.70 (1.54-1.85)	1.69 (1.52-1.82)	.518
NYHA class ≥III	19 (6)	12 (5)	7 (6)	.562
Hypertension	138 (40)	99 (45)	39 (32)	.029
Diabetes mellitus	27 (8)	19 (9)	8 (7)	.675
Dyslipidemia	122 (36)	90 (41)	32 (26)	.010
Chronic kidney disease	25 (7)	20 (9)	5 (4)	.128
COPD	15 (4)	12 (5)	3 (2)	.274
Atrial fibrillation	27 (8)	17 (8)	10 (8)	.836
History of stroke	17 (5)	10 (5)	7 (6)	.610
Etiology				>.99
Aortic stenosis	189 (55)	122 (55)	67 (55)	
Aortic regurgitation	154 (45)	100 (45)	54 (45)	
Left ventricular				
Ejection fraction	56 (47-62)	58 (48-62)	54 (47-60)	.110
End-diastolic diameter	57 (47-66)	56 (46-65)	58 (49-69)	.104
End-systolic diameter	38 (28-48)	37 (28-48)	39 (30-49)	.363
Left atrial diameter, mm	39 (34-46)	39 (34-45)	40 (35-48)	.157

Data are presented as mean ± SD, n (%), or median (interquartile range).

COPD, chronic obstructive pulmonary disease; NYHA, New York Heart Association Functional Classification.

Surgical data and early outcomes are summarized in Table 2. SAVR was performed using a median sternotomy in 290 patients (85%) or a right minithoracotomy in 53 patients (15%).

**Table 2:** Surgical Data and Early Outcomes

Variables	Total	Bioprosthetic Valve	Mechanical Valve	*P* Value
	(N = 343)	(n = 222)	(n = 121)	
Approach				.274
Sternotomy	290 (85)	184 (83)	106 (87)	
Right minithoracotomy	53 (15)	37 (17)	16 (13)	
Concomitant surgery				
Coronary artery bypass grafting	15 (4)	10 (5)	5 (4)	>.99
Mitral valve repair	11 (3)	7 (3)	4 (3)	>.99
Mitral valve replacement	15 (4)	7 (3)	8 (7)	.170
Graft replacement of ascending aorta	52 (15)	43 (19)	9 (7)	.002
Maze procedure	19 (6)	14 (6)	5 (4)	.466
Operation time, min	279 ± 76	274 ± 78	288 ± 72	.100
Cardiopulmonary bypass time, min	142 ± 45	139 ± 45	147 ± 45	.134
Cardiac arrest time, min	102 ± 34	101 ± 35	104 ± 33	.327
Stroke	1 (0.3)	0	1 (0.8)	>.99
Length of stay				
Intensive care unit, d	2.0 ± 1.2	2.1 ± 1.3	1.7 ± 1.0	<.01
Hospital, d	13.4 ± 5.7	12.6 ± 6.0	14.8 ± 4.8	<.01
In-hospital mortality	1 (0.3)	0	1 (0.8)	>.99

Data are presented as n (%) or mean ± SD.

### Early Outcomes

Postoperative stroke occurred in 1 patient after mechanical AVR. There was 1 in-hospital death in the mechanical AVR group caused by intestinal ischemia. The 30-day and in-hospital mortality rates in the entire cohort were 0.3%.

### Survival Rate

During follow-up, 15 deaths (9 in the bioprosthetic AVR group vs 6 in the mechanical AVR group) occurred, and the overall 5-, 10-, and 15-year survival rates in the entire cohort were 98.2%, 92.4%, and 87.4%, respectively. The 5-, 10-, and 15-year survival rates were 97.5%, 91.0%, and 87.6%, respectively, in the bioprosthetic AVR group and 99.2%, 94.1%, and 88.6%, respectively, in the mechanical AVR group (log-rank *P* = .384). The difference in the survival rate between patients undergoing SAVR (both prosthetic and mechanical AVR) and the age- and sex-matched Japanese general population was not significant (log-rank *P* = .899) (Figure panel A). This result was unaffected when patients with SAVR were stratified by age ≤60 years (log-rank *P* = .563) and age ≤50 years (log-rank *P* = .657) (Figure panels B and C). The standardized mortality ratios in the entire cohort (≤65 years old) and in patients ≤60 and ≤50 years old were 1.033 (95% CI, 0.584-1.829), 1.213 (95% CI, 0.584-2.518), and 1.367 (95% CI, 0.320-5.849), respectively (Table 3).

**Figure:**
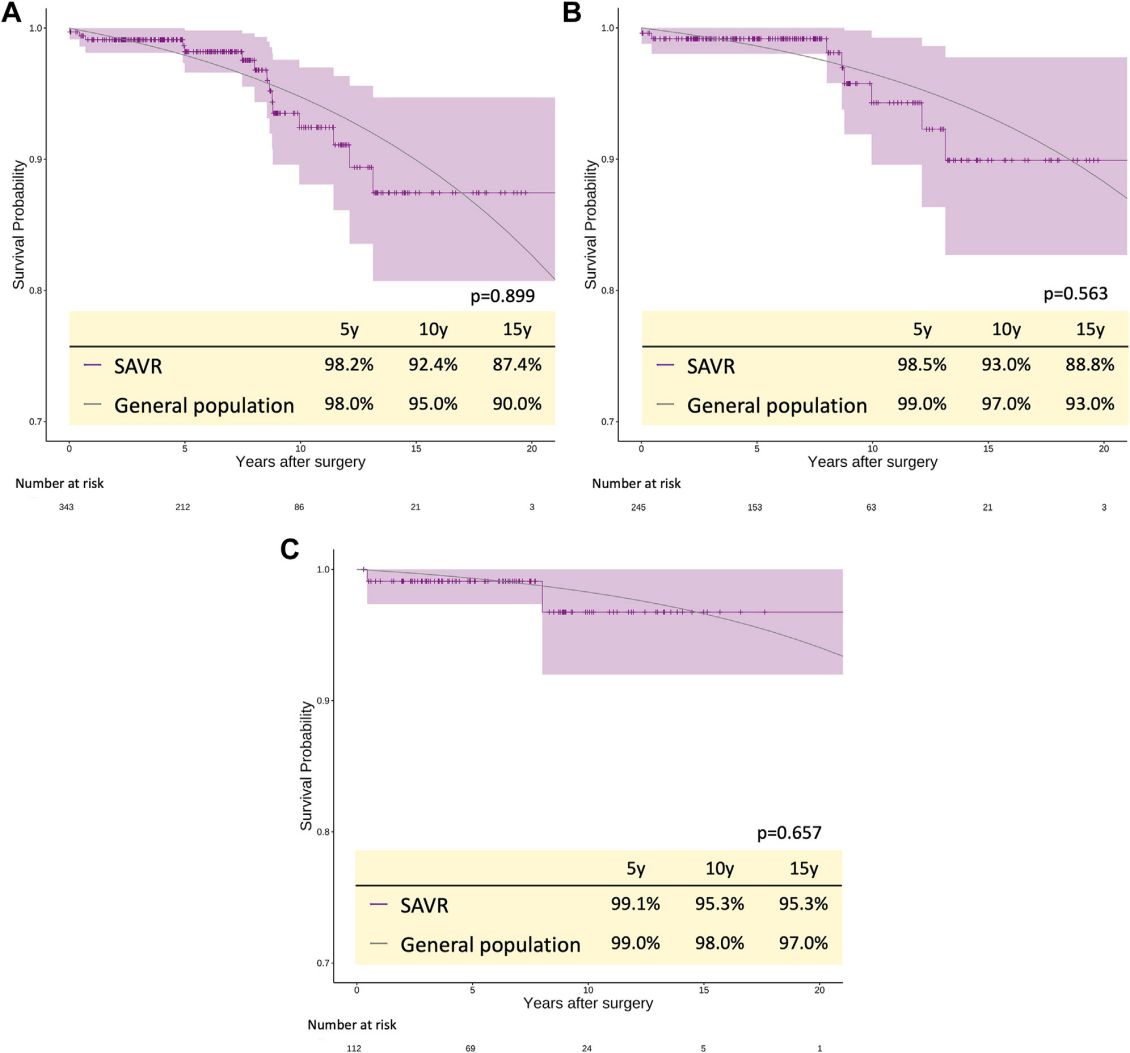
Survival rates of patients after surgical aortic valve replacement (SAVR) with bioprosthetic or mechanical aortic valve compared with an age- and sex-matched Japanese general population. (A) All study population, (B) patients aged ≤60 years, and (C) patients aged ≤50 years. The shaded areas indicate the 95% CI.

**Table 3:** Standardized Mortality Ratio

Variable	All Study Population	Patients ≤60 Years	Patients ≤50 years
	(N = 343)	(n = 245)	(n = 112)
Age, y	53.2 ± 10.5	48.8 ± 9.7	40.7 ± 7.5
Log-rank *P* value	.899	.563	.657
Standardized mortality ratio	1.033 (0.584-1.829)	1.213 (0.584-2.518)	1.367 (0.320-5.845)

Data are presented as mean ± SD or median (interquartile range).

### Valve-Related Events During Follow-up

#### Reoperation

**Supplementary Figure 1:**
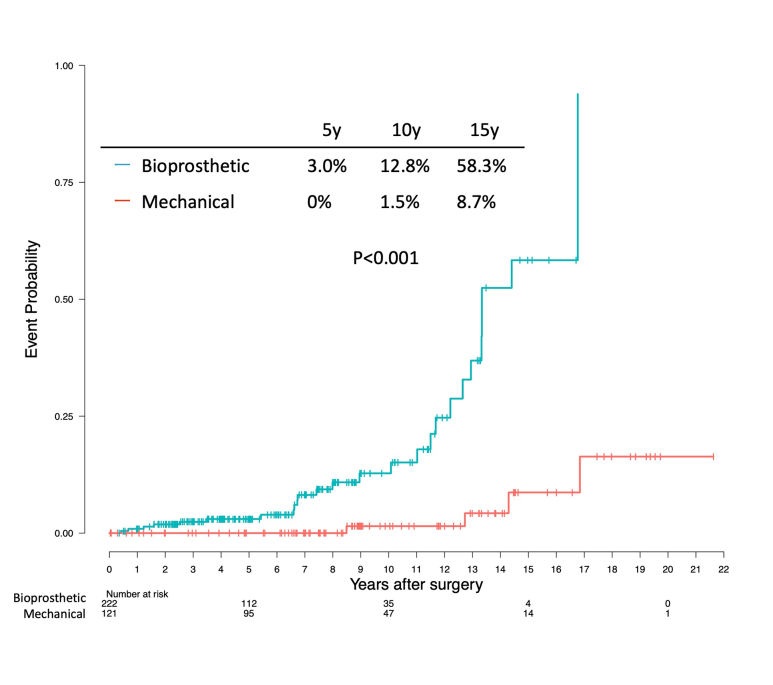


A total of 30 reoperations were performed (26 in the bioprosthetic AVR group and 4 in the mechanical AVR group) for prosthetic valve failure (n = 24) or prosthetic valve endocarditis (n = 6). The 5-, 10-, and 15-year cumulative incidences of reoperation were 3.0%, 12.8%, and 58.3% in the bioprosthetic AVR group and 0%, 1.5%, and 8.7%, respectively, in the mechanical AVR group (log-rank *P* < .001) (Supplemental Figure 1).

#### Stroke

**Supplementary Figure 2:**
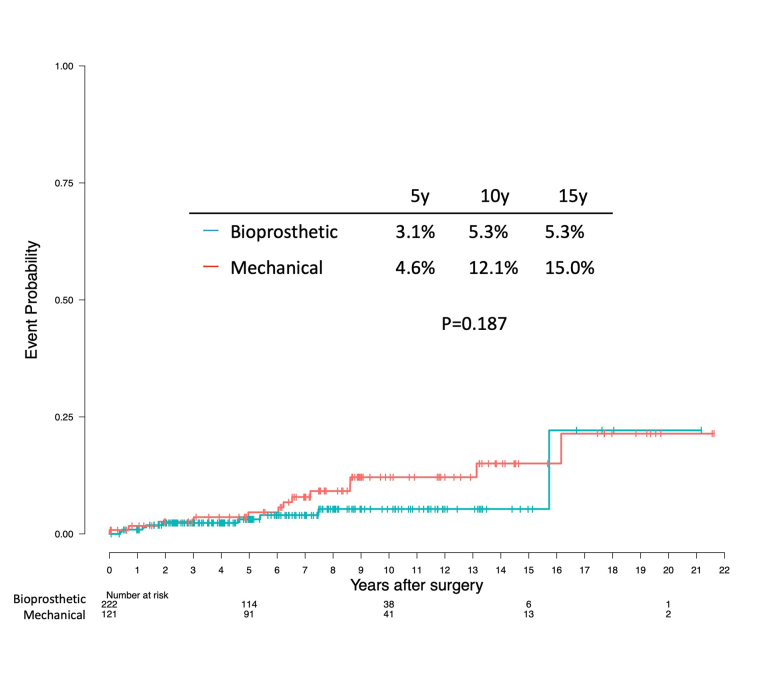


There were 23 strokes, consisting of 16 cardiogenic embolism events (9 in the bioprosthetic AVR group and 7 in the mechanical AVR group) and 7 intracranial bleeding events (2 in the bioprosthetic AVR group and 5 in the mechanical AVR group). The 5-, 10-, and 15-year cumulative incidences of stroke were 3.1%, 5.3%, and 5.3%, respectively, in the bioprosthetic AVR group, and 4.6%, 12.1%, and 15.0%, respectively, in the mechanical AVR group (log-rank *P* = .187) (Supplemental Figure 2).

#### Endocarditis

Endocarditis occurred in 10 patients (8 with a bioprosthetic AVR and 2 with a mechanical AVR); of these, 6 underwent reoperation, and the remaining 4 were treated medically. The 5-, 10-, and 15-year cumulative incidences of endocarditis were 3.5%, 5.0%, and 5.0%, respectively, in the bioprosthetic AVR group, and 0%, 1.9%, and 4.6%, respectively, in the mechanical AVR group (log-rank *P* = .136) (Supplemental Figure 3).

## Comment

This retrospective observational study aimed to clarify the long-term clinical outcomes after SAVR in young adults aged ≤65 years. The survival rate of patients aged ≤65 years after SAVR was equivalent to that of the age- and sex-matched Japanese general population.

Compared with our report of equivalent survival rates after SAVR in Japanese patients aged ≤65 years with those in the general Japanese population, El-Hamamsy and colleagues^5^ recently reported that survival rates after mechanical or biological SAVR were markedly worse than those in the general population in the United States, irrespective of the type of prosthetic valve. This discrepancy can be explained by several factors. First, with regards to the patient selection, the United States study enrolled young adults aged 18 to 50 years, whereas ours enrolled patients aged ≤65 years, leading to a substantial difference in the mean age (36 years vs 53.2 years). This substantial difference in patient age may suggest that patients in the United States were more likely to require a repeat aortic valve procedure and, therefore, present with valve-related morbidities, which might have negatively affected survival. The survival rate at 15 years in our cohort was 87.6% in the bioprosthetic AVR group and 88.6% in the mechanical AVR group, which was not inferior to those in the United States study (87.9% in the bioprosthetic AVR group and 88.4% in the mechanical AVR group), although the mean age of our cohort was ∼17 years older than that in the United States study. When we focused on patients aged ≤50 years (n = 112), as in the United States study, the mean age became 40.7 years and the survival rates at 15 years were 91.6% in the bioprosthetic AVR group and 100% in the mechanical AVR group, which were higher than those in the United States study. The relatively better survival rates of our cohort might have led to a comparable survival rate between our cohort and the age- and sex-matched Japanese general population.

### Clinical Implications

This study showed that the survival rate after SAVR of patients aged ≤65 years was equivalent to that of the age- and sex-matched Japanese general population, which supports the concept that SAVR is the gold standard treatment for patients aged ≤65 years who need aortic valve surgery. This finding is also supported by the current guidelines for heart valve diseases.^7^^,^^8^ The survival rates of our patients aged ≤60 years (mean age, 48.8 years) or ≤50 years (mean age, 40.2 years) were not inferior to those of the age- and sex-matched Japanese general population, but the standardized mortality ratios were 1.083 in the entire cohort (≤65 years), 1.213 in patients ≤60 years, and 1.367 in patients ≤50 years, respectively. Although these *P* values did not reach statistical significance, likely because of the small sample size, our data suggest that the difference in survival rate between the 2 groups can be affected by the age at the time of AVR, and the greater the difference in the long-term mortality rate, the younger are the patients undergoing AVR. The increased standardized mortality ratio in younger patients observed in our study is not inconsistent with that reported in the United States study; thus, further research, prompted by our findings, should be done to validate the superiority of the Ross procedure in young adults in Japan.

### Limitations

The present study has some limitations that should be acknowledged. First, this was a retrospective study with a relatively small sample size. In addition, we excluded patients aged >65 years and those who had a history of aortic valve surgery or with active endocarditis to mitigate potential biases and restricted our analysis to only those ≤65 years with aortic valve disease. Second, although this cohort included patients who underwent concomitant operations, these additional operations did not significantly impact mortality. Finally, longer follow-up is necessary to determine the optimal valve substitute for this population.

### Conclusions

The survival rate of young adults aged ≤65 years who underwent SAVR was equivalent to that of the age- and sex-matched general Japanese population. Our data may help guide future therapeutic comparisons for patients aged ≤65 years requiring aortic valve procedures.

## Figures and Tables

**Supplementary Figure 3:**