# Supplementary material for: Long-term Outcomes After Surgical Aortic Valve Replacement in Patients ≤65 Years: Comparison of Age- and Sex-Matched Japanese General Population
Source: Ann Thorac Surg Short Rep. 2025 Jan 3;3(2):432–7. doi: 10.1016/j.atssr.2024.12.008 (PMC12167536; doi:10.1016/j.atssr.2024.12.008)
Supplement: Legends for Supplementary Figures [file mmc1.docx]

**SUPPLEMENTAL FIGURE LEGENDS**

**Supplemental Figure 1.** Cumulative incidence of reoperation after SAVR with bioprosthetic or mechanical valve. SAVR, surgical aortic valve replacement.

**Supplemental Figure 2.** Cumulative incidence of stroke after SAVR with bioprosthetic or

mechanical valve. SAVR, surgical aortic valve replacement.

**Supplemental Figure 3.** Cumulative incidence of endocarditis after SAVR with bioprosthetic or mechanical valve. SAVR, surgical aortic valve replacement.
