# Supplementary material for: Long-term Outcomes After Surgical Aortic Valve Replacement in Patients ≤65 Years: Comparison of Age- and Sex-Matched Japanese General Population
Source: Ann Thorac Surg Short Rep. 2025 Jan 3;3(2):432–7. doi: 10.1016/j.atssr.2024.12.008 (PMC12167536; doi:10.1016/j.atssr.2024.12.008)
Supplement: Supplementary Material [file mmc2.docx]

**Patients and Methods**

This was a non-randomized retrospective study encompassing patients aged ≤65 years who underwent primary SAVR with or without concomitant coronary artery bypass grafting, mitral surgery, or ascending aortic surgery at our center between 2001 and 2021. Patients who had a history of aortic valve surgery and required a second AVR, or those with active infectious endocarditis were excluded. SAVR was performed with either a bioprosthetic or mechanical prosthetic valve after careful discussion with the patients based on the Japanese guidelines on the management of valvular heart diseases. SAVR was performed through a median sternotomy or right mini-thoracotomy with a tepid hypothermic cardiopulmonary bypass.

The study complied with the principles outlined in the Declaration of Helsinki, and was approved by the Institutional Review Board of the National Cerebral and Cardiovascular Center (reference number: M30-026). All patients provided informed consent before undergoing the procedures.

**Clinical endpoints and follow-up**

The primary endpoint was the all-cause mortality during follow-up, and we compared with that of an age- and sex-matched Japanese general population. The patients who underwent SAVR were stratified by age: ≤60 years and ≤50 years.

**Statistical analysis**

No special treatment was performed for missing data or outliers, and the observed data were used as they were gathered. Continuous variables were expressed as median and interquartile range, and categorical variables were expressed as numbers and frequencies (percentages). Comparisons between groups were evaluated using the Mann–Whitney U test for continuous variables, and Fisher's exact test or chi-square test for categorical variables, as appropriate.

To analyze the primary and secondary events, we estimated the expected number of events for each event in the general population of Japan, corresponding to age and sex. Based on these estimates, we constructed survival curves for the general population and calculated standardized mortality ratios. Survival curves for the general and study populations were compared using the Finkelstein–Muzikansky–Schoenfeld one-sample log-rank test. The life table data on survival rates for the general population were obtained from the official website of the Ministry of Health, Labour, and Welfare of Japan (https://www.mhlw.go.jp).

To investigate the differences in trends by age, similar analyses were performed on age subpopulations (≤60 and ≤50 years). All p-values were two-tailed, with 95% confidence intervals (CI). All statistical analyses were conducted using the R software version 4.3.1.
